# Metabolic Impacts of Confinement during the COVID-19 Pandemic Due to Modified Diet and Physical Activity Habits

**DOI:** 10.3390/nu12061549

**Published:** 2020-05-26

**Authors:** María Martinez-Ferran, Fernando de la Guía-Galipienso, Fabián Sanchis-Gomar, Helios Pareja-Galeano

**Affiliations:** 1 Faculty of Sports Sciences and Physiotherapy, Universidad Europea de Madrid, Villaviciosa de Odón, 28670 Madrid, Spain; maria.martinez.nutricion@gmail.com; 2 Cardiology Service, Hospital Clínica Benidorm, Benidorm, 03501 Alicante, Spain; fdelaguia@gmail.com; 3 Glorieta Policlinic, Dénia, 03700 Alicante, Spain; 4 REMA Sports Cardiology Clinic, Denia, 03749 Alicante, Spain; 5 Department of Physiology, Faculty of Medicine, INCLIVA Biomedical Research Institute, University of Valencia, 46010 Valencia, Spain; fabian.sanchis@uv.es; 6 Division of Cardiovascular Medicine, Stanford University School of Medicine, Stanford, California, CA 94305, USA

**Keywords:** COVID-19, acute sedentary lifestyle, step reduction, positive energy balance, metabolic consequences, insulin resistance, metabolic syndrome, sarcopenia

## Abstract

While the detrimental effects of a chronic positive energy balance due to a sedentary lifestyle have been well established, the impacts of a short period of abruptly reduced physical activity and overeating arising from strict confinement due to the COVID-19 pandemic will soon start to emerge. To reasonably anticipate major consequences according to the available evidence, we hereby review the literature for studies that have explored the health impacts of several weeks of a reduction in physical activity and daily step-count combined with modified eating habits. These studies identify as main metabolic consequences increases in insulin resistance, total body fat, abdominal fat and inflammatory cytokines. All these factors have been strongly associated with the development of metabolic syndrome, which in turn increases the risk of multiple chronic diseases. A plausible mechanism involved in these impacts could be a positive energy balance promoted by maintaining usual dietary intake while reducing energy expenditure. This means that just as calorie intake restriction could help mitigate the deleterious impacts of a bout of physical inactivity, overeating under conditions of home confinement is very likely to exacerbate these consequences. Moreover, hypertension, diabetes, and cardiovascular disease have been identified as potential risk factors for more severely ill patients with COVID-19. Thus, adequate control of metabolic disorders could be important to reduce the risk of severe COVID-19.

## 1. Introduction

The current COVID-19 pandemic has led governments of the mainly affected countries to impose strict confinement rules on their citizens. These include measures such as working from home and closing schools, shops, restaurants and any business or service considered non-essential in order to slow down the spread of the contagion and thereby prevent the collapse of health care systems. These measures have, however, had their impacts on the general health of the population because of both exercise restrictions and effects on diet. Exercise restrictions have been the consequence of closed gyms and sports centers, restrictions on walking distance, lack of space and infrastructure of homes for physical exercise, and lack of technical knowledge of the population on appropriate training routines. Effects on nutrition include limited access to shops, a poorer quality of food products due to the already visible impacts on family income, and overeating. Before this pandemic, insufficient physical inactivity was already described as a global public health problem, with over a quarter of all adults not undertaking the levels of physical activity required for good health [1]. As a result of the current situation in which many people are confined to their homes, physical activity and exercise levels drastically decline while dietary habits remain unchanged or fail to offset this inactivity producing a positive energy balance. There is strong epidemiological evidence that a chronic sedentary lifestyle is detrimental for health [2,3]. Likewise, there is evidence that such negative effects persist even when performing physical exercise programs, revealing that it is just as important to reduce sitting times as it is to lengthen exercising periods [4].

Exercise plays a fundamental role in the prevention of most chronic diseases. Our body needs a relatively long period to benefit from the healthy adaptations that exercise generates, modulated by different molecular mechanisms such as epigenetics, metabolic modulation or reduced inflammation [5,6,7,8,9,10,11,12,13]. Unfortunately, it requires only a period of a few days to reverse these adaptations, and the body returns to a physiological situation similar to baseline or even worse [2]. This means that trying to maintain an active lifestyle during home quarantine is essential to avoid physical consequences and this approach may also help mitigate the psychological impacts of confinement, especially among the elderly [14,15,16].

**Figure 1:**
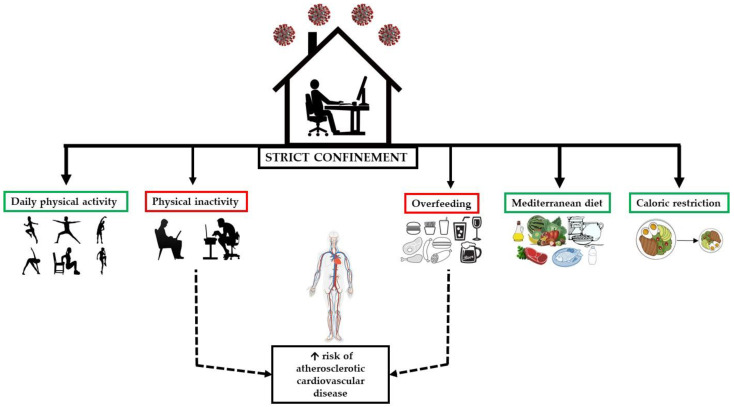
Consequences of overfeeding and reduced physical activity.

To analyze the consequences of physical inactivity and an acute positive energy balance due to changes in eating habits, different models have been employed [17,18]. Nevertheless, these interventions do not reflect the current home confinement situation, in which daily physical activity is drastically reduced and there is a tendency to eat more and worse affecting the risk of metabolic-associated chronic diseases such as cardiovascular diseases (CVD) across a large part of the world population (Figure 1). This paper therefore reviews evidence of the metabolic-health impacts of a short period of a reduction in physical activity and a tendency to overeat.

## 2. Methods

Electronic databases (Medline, EMBASE, and Web of Science) were searched without language restrictions to identify all reports on metabolic-related alterations, physical inactivity and overfeeding. Inclusion criteria were: (i) publication in a peer-reviewed journal, (ii) human study, (iii) studies examining the impacts of acute physical inactivity, and (iv) studies examining the impacts of acute changes in dietary habits. Reasons for exclusion were: (i) studies in non-adult subjects, (ii) no control group, (iii) data reported not usable.

## 3. Results and Discussion

### 3.1. Metabolic Consequences of Acute Physical Inactivity in Healthy Adults

In this section, we review the impact of acute physical inactivity on glycemic control, inflammatory markers, body composition and cardiorespiratory fitness (CRF) in healthy young adults (Table 1).

Pedersen et al. [19,20] employed a step reduction model to examine the consequences of current lifestyles involving prolonged periods of inactivity interspersed with short episodes of low to moderate physical activity. In their investigation, participants were instructed to reduce daily steps by taking lifts instead of stairs and using their cars instead of walking or cycling. 

The two following studies were conducted on healthy young men who performed less than 2 h of regular exercise per week and walked more than 3500 steps per day. During the interventions, participants maintained their usual dietary habits. In the first study, participants were recruited for 2 sub-studies [19]. In the first sub-study [19], eight participants (27.1 (5.7) years; body mass index (BMI) 22.9 (4.0) kg/m^2^) reduced their activity from 6203 steps/day (5135–7271) to 1394 steps/day (1261–1528) for three weeks. Results included a significant increase in the area under the curve (AUC) for plasma insulin during an oral glucose tolerance test (OGTT) from baseline to the third week. In the second sub-study of the first study [19], ten subjects (23.8 (4.6) years; BMI 22.1 (2.1) kg/m^2^) reduced their mean activity level of 10,501 steps/day (8755–12,247) to 1344 steps/day (1272–1416) for 2 weeks. In this study, both plasma insulin AUC and plasma C-peptide levels increased significantly after the inactivity period. In the oral fat tolerance test, AUCs for plasma insulin, C-peptide and triglycerides (TG) also increased significantly. Further, while total fat mass (FM) remained unchanged, intra-abdominal FM increased (by 7%) after the two weeks of step reduction, and this was accompanied by a significant reduction in total free fat mass. In the second study [20], ten young healthy males (23.8 (1.5) years; BMI 22.1 (0.7) kg/m^2^) reduced their daily steps from a baseline value of 10,501 (808) to 1344 (33) for two weeks. This step reduction led to a decreased glucose infusion rate due to a reduction in peripheral insulin sensitivity, and a concurrent decrease in insulin-provoked muscle Akt phosphorylation with no effect on endogenous glucose production in the liver. In contrast to the results of study 2 [19], there was no significant change in total FM after step reduction, but leg lean mass was lower. Finally, two weeks of physical inactivity did not produce changes in plasma levels of TG, free fatty acids (FFA), glucose, insulin or C-peptide.

Interestingly, the findings of a similar study indicated that just three days of step reduction from 12,956 (769) to 4319 (256) steps/day led to impaired glycemic control in twelve healthy active participants (8 men, 4 women; 29 (1) years; 23.6 (0.9) kg/m^2^) [21]. In response to this short intervention, the authors also noted increased postprandial glucose levels, increased fasting plasma insulin and C-peptide responses to OGTT, along with increased insulin resistance and diminished insulin sensitivity.

Maximal aerobic capacity (VO_2max_) is a major predictor of functional capacity and is the gold standard indicator of CRF. A person’s VO_2max_ also reflects pulmonary and muscle function, nutritional status or the health state of other organ systems. While higher CRF is associated with better health, lower CRF is associated with increased mortality independently of other risk factors [2]. Several studies [20,22,23,24] have determined the effects of an acute period of inactivity on VO_2max_. Results indicated that following two weeks of physical inactivity VO_2max_ was reduced when participants maintained their normal diet [20,24], when calorie intake was restricted [23] or when intake was increased by 50% kcal [22]. In two of these studies, nevertheless, it was found that VO_2max_ returned to baseline after normal physical activity was resumed [22,24].

Collectively, the above data indicate that a reduction in daily physical activity of three days, two weeks or three weeks impairs glycemic control. In addition, two weeks of step reduction increased FM and reduced lean mass, and also had a negative impact on VO_2max._

### 3.2. Metabolic Consequences of Acute Physical Inactivity in Overweight Adults

In the previous articles, interventions were conducted on subjects within the normal weight range. Additionally, similar step reduction interventions have been tested on both overweight and normal weight populations to compare the consequences of acute physical inactivity (Table 1).

To examine whether reduced physical activity in adults with central overweight could lead to a rapid decline in metabolic and inflammatory homeostasis compared to what happens in lean counterparts, Dixon et al. [25] performed a study in which individuals were subjected to a week of step reduction to below 4000 steps/day. The participants of this study were nine active men with central overweight (49 (1.0) years; BMI 29.3 (1.2) kg/m^2^) and nine active lean men (51.5 (1.4) years; BMI 29.3 (1.2) kg/m^2^) who maintained their normal diet. In the former group, subjects had significantly higher total FM and abdominal FM levels before the intervention (these variables were not nevertheless determined after the intervention). Results indicated that, while insulin and glucose AUC responses to an OGTT and fasting TG concentrations increased in both groups, in the overweight group, glucose and insulin AUC, TG and C-reactive protein (CRP) and alanine transaminase were all higher before the intervention and remained so throughout. No changes were produced in total cholesterol, low-density lipoprotein (LDL) or high-density lipoprotein (HDL) cholesterol over the intervention period and neither did differences emerge between groups.

In another study, the metabolic consequences of a drop in physical activity from a daily step-count of <10,000 to 1500 for 14 days were examined in 45 active healthy participants who continued with their usual diet [24]. Of these 45 participants, 16 had first-degree relatives with type 2 diabetes (10 females, 6 males; 40 (14) years; BMI 27 (5) kg/m^2^) and 29 did not (18 females, 11 males; 33 (13) years; BMI 24 (3)). Those in the former group had a significantly higher BMI classified as “overweight” and greater waist and hip circumferences, although there were no significant differences in FM. Both groups experienced a significant reduction in insulin sensitivity accompanied by a significant decrease in glucose and insulin AUC. Although both groups showed a reduction in muscle insulin sensitivity, the overweight group displayed a lower sensitivity. The period of reduced physical activity significantly lowered VO_2max_ across the study population without differences between groups. Total lean mass and lower limb lean mass decreased significantly and there was a significant increase in total FM and liver fat; those with overweight accumulated more android fat (1.5%) after step reduction. Lipid profiles were also modified in that higher total cholesterol, LDL-cholesterol and TG were recorded after step reduction, a greater TG increase being detected in the overweight subjects. All variables returned to baseline values 14 days after the subjects resumed their usual physical activity. After resuming normal activity, the overweight group engaged in lower amounts of vigorous activity and had lower insulin sensitivity.

According to the findings of a study by Bowden et al. [29], obese individuals with metabolic syndrome had lower CRF (measured as VO_2max_ peak) than both non-obese subjects without metabolic syndrome and non-obese individuals with metabolic syndrome, the latter being the most sedentary population. The authors also found that higher VO_2max_ peak, lesser sedentary time and average daily METS were correlated with lower liver fat. Results suggested that high levels of CRF in the overweight and obese population significantly reduced or eliminated the elevated risk of CVD and all-cause mortality. This indicates that CRF changes the relationship between body fat and its prognosis. We should underscore that many of the benefits of improved CRF are derived from an increase in physical activity [30].

In conclusion, in these studies a reduction in acute physical activity negatively influenced glycaemia control and lipid profile (TG, total cholesterol, LDL-cholesterol). Regarding body composition, step reduction increased FM, liver fat mass and reduced lean mass in both overweight and normal weight subjects. However, in those overweight, consequences were usually somewhat greater.

### 3.3. Detrimental Health Effects of an Acute Sedentary Lifestyle in the Elderly

The prevalence of sarcopenia is high among the elderly. A loss of skeletal muscle mass and strength has several repercussions on health, and all conditions in which muscle activity is reduced can lead to sarcopenia [31]. Moreover, ageing is associated with abdominal obesity, an important contributor to insulin resistance and metabolic syndrome, along with a higher level of proinflammatory cytokines [32]. Accordingly, drastic decreases in physical activity could have worse consequences in elderly subjects by accelerating the ageing process and the appearance of age-related diseases. As an example, the two studies described below examine the impacts of a step reduction intervention in elderly subjects on glycemic control, body composition, inflammatory parameters and CRF (Table 1).

Breen et al. (2013) conducted a study in healthy older adults (5 males, 5 females; 72.3 (1.0) years; BMI 29.0 (1.8)) who were moderately active (>3500 steps/day). During the intervention, participants reduced their daily step-count by approximately 76% of habitual levels while maintaining their dietary habits. After this period, insulin resistance was increased and postprandial insulin sensitivity was reduced. Fasting insulin concentrations and its peak plasma concentrations at 30 min of OGTT were greater after step reduction and AUC for plasma glucose and insulin during OGTT increased. The body composition data revealed that after step reduction, body fat percentage increased and skeletal leg muscle mass was significantly reduced. Further findings were postprandial rates of myofibrillar protein synthesis reduced by approximately 26% after the intervention with no difference in postabsorptive rates.

A similar step-reduction intervention was conducted in 22 moderately active older adults (12 males: 69 (3) years, BMI 27.3 (4.6): 10 females; 70 (5) years, BMI 27.7 (5.1)) [28]. Participants reduced their daily step-count by 70% and maintained their usual dietary habits. Body composition variables remained unchanged. However, the authors reported that just a week of step reduction led to increased insulin resistance and reduced insulin sensitivity. Moreover, glucose and insulin AUC were elevated as were fasting plasma glucose and insulin concentration during OGTT. Also observed was a reduction in muscle protein synthesis. What it is interesting to point out is that after the step reduction protocol, participants were reassessed after 14 days of return to their habitual step-count. In this examination it was confirmed, however, that glycemic control and inflammatory markers had not recovered. In contrast, in a younger study population (36 (14) years) changes in metabolic variables produced were reversed when normal physical activity levels were recovered [28]. 

In both studies conducted in older subjects, plasma concentrations of inflammatory markers (TNF-α and CRP) were significantly increased after the step reduction intervention [27,28]. In the second study [28], IL-6 was also increased and after returning to normal activity, inflammatory markers were still elevated. In contrast, in the studies conducted in young individuals, no changes were produced in inflammatory parameters [20,22,25,26].

In summary, acute physical inactivity led to impaired glycemic control, increased inflammation and reduced muscle protein synthesis. Inactivity may also reduce fat free mass while increasing FM. In addition, recovering normal levels of activity in the elderly could be harder compared to younger subjects.

### 3.4. Metabolic Effects of Acute Physical Inactivity plus Overfeeding

Energy balance is the state in which energy intake equals energy expenditure. A positive energy balance, whereby energy intake exceeds expenditure, can lead to weight gain due to increased body fat [33]. In the studies described in the previous section, participants kept up their usual dietary intake while reducing energy expenditure, resulting in a positive energy balance that may have contributed to the observed metabolic changes [19,20,24,25]. In this section, we review the impact of physical inactivity when added to a diet intervention (Table 1).

In a study of the effects of two weeks of step reduction combined with overfeeding, nine healthy young men (24.3 (3.3) years; BMI 21.6 (2.5) kg/m^2^) undertook 14 days of step reduction from 10,278 (2399) to 1521 (488) steps/day and increased their daily total energy intake by 50% kcal [22]. This study showed that insulin sensitivity reduction occurs after three days of inactivity and overfeeding. Clamp-derived insulin sensitivity was reduced after 14 days of inactivity and oral glucose tolerance remained unaffected. The insulin response to OGTT increased after the first and the second week. Body composition was affected by the intervention, in that body weight was higher due to an increase in total FM (1.5 (0.5) kg; *p* < 0.05), and in android, gynoid and visceral fat. Finally, plasma levels of leptin and adiponectin increased after the intervention and no changes in TG or FFA were observed. The authors of this study also reported that after 16 days of returning to an uncontrolled free-living environment, body weight and adiposity were still elevated while remaining variables returned to baseline values.

Interestingly, one study explored whether metabolic dysfunction caused by inactivity might be blunted by energy restriction [23]. Ten physically active men and women (24 (1) years) reduced their daily steps from 10,000 to 5000 for 10 days. Participants completed two periods of physical inactivity while consuming either a control diet (16% kcal from protein, 64% kcal from carbohydrate, 20% kcal from fat) or a higher-protein diet (30% kcal from protein, 50% kcal from carbohydrate, and 20% kcal from fat) in a randomized crossover design. In both diets, energy intake was decreased by 15–20% of total energy expenditure to offset the reduction in energy expenditure (400 kcal/day). As a positive control condition, a group of subjects from the initial sample (n = 5) repeated the same protocol of inactivity in association with overfeeding (35% kcal). The results of this study revealed that when diet was controlled, body fat was not altered by physical inactivity and body weight was significantly reduced; abdominal FM was also lowered. In contrast, when overfeeding was accompanied by inactivity, both body weight and body fat went up. Further, when overfeeding was combined with inactivity, the authors observed increases in fasting blood glucose, plasma insulin, plasma c-peptide, insulin resistance, and 2 h postprandial glucose and insulin concentrations. However, when the diet was controlled none of these changes were produced.

Other authors have looked at what happens when a reduction in physical activity and overfeeding are accompanied by an exercise intervention [26]. Over one week, 26 physically active men (25 (7) years; BMI 23.8 (2.5)) were randomly assigned to two groups. In both groups, physical activity was restricted to under 4000 steps/day and energy intake increased (50% kcal) while individuals in one of the groups undertook 45 min of daily treadmill running at 70% of VO_2max_. In both groups, increases were recorded in body weight, waist/hip circumference and lean mass. In the group of subjects who did not train, insulin sensitivity and B-cell function were reduced and the insulin response to OGTT was increased. This group also showed an increase in total cholesterol and adiponectin. However, the addition of physical exercise was able to abolish these changes.

Taken together, these findings indicate that energy balance plays a key role in the metabolic consequences of acute physical inactivity. Accordingly, while overeating could worsen its repercussions, energy restriction could help avoid its impacts. Although physical exercise seems to improve glycemic control, a positive energy balance still affects body composition. In the studies reported, however, calorie intake was controlled, so it is unknown whether reduced physical activity may have led to reduced energy intake.

Physical activity plays an important role in energy balance, and subjects engaging in higher levels of physical activity may have improved sensitivity of the appetite control system [34]. Evidence shows that there is weak coupling between energy intake and expenditure in individuals displaying low levels of daily physical activity, but strong coupling with high levels of physical activity [35].

In 1956, Mayer [36] described that calorie intake increases with activity only within a certain zone of “normal activity”. This author also confirmed that below a level of physical activity or so-called “sedentary zone”, a further decrease in activity is not followed by a decrease in food intake. Recent investigations have also shown that a reduction in physical activity is not usually offset by a reduction in energy intake, resulting in a positive energy balance [37,38]. Further, it has been proposed that sedentary activities not only reduce energy expenditure, but they also promote increased food intake. Hence activities such as watching television or cognitive tasks stimulate food intake such that the sensations of satiety and fullness are ignored leading to overconsumption [39]. This evidence suggests it is highly likely that a significant reduction in physical activity will not be accompanied by a reduction in energy intake and this will result in a positive energy balance likely worsening the metabolic effects of sedentary behavior.

### 3.5. Manipulating Dietary Intake to Offset the Metabolic Impacts of Confinement

While it is important to remain active to avoid the problems an acute sedentary lifestyle brings, what about our attitude to food during this period of confinement? On the one hand, there may be overfeeding, while on the other, calorie intake may be restricted due to reduced activity or physical inactivity. Restricted calorie intake could be an optimal option for our current situation. This means a diet lower in a given percentage of calories than our regular diet, but that, nevertheless, is balanced to include all the necessary nutrients. Research in some animals has shown that the intake of up to 40% fewer calories has an impressive positive effect on markers of disease and ageing [18]. In humans, a few randomized controlled clinical trials have examined the effects of calorie restriction on health. The findings of a study performed in healthy subjects who underwent a three-month period of calorie restriction, i.e., five consecutive days per week of a fasting-mimicking diet low in calories, proteins, and sugars but high in unsaturated fatty acids, were reduced BMI, trunk, and total body fat [40]. Likewise, reductions were recorded in blood pressure, triglycerides, total and low-density lipoprotein cholesterol, C-reactive protein, and insulin-like growth factor 1 (IGF-1). The authors concluded that cycles of a five-day fasting-mimicking diet were safe, feasible, and effective in reducing risk factors associated with metabolic-related diseases.

In a two-year follow-up clinical trial, the effect of two years of 15% calorie restriction was assessed in healthy individuals. Results indicated an average weight loss of 8.7 kg (70% was body fat) in the calorie restriction group versus an average gain of 1.8 kg in the control group. Further, subjects in the restricted calorie intake group showed a 10% reduction in the metabolic rate of sleep, associated with reduced levels of reactive oxygen species and thyroid activity (reduced T3 and T4), which are biomarkers of aging [41]. In another study carried out in eighteen healthy and physically active subjects, the effects of caloric restriction close to 40% of the standard calorie intake for six weeks were assessed [42]. Diet was based on three days of severe restriction (600–800 Kcal) per week and normal intake for the rest of the week. Results indicated considerable weight loss including reduced fat mass (mostly android) and a less appreciable effect on fat-free mass. Hence, the option of calorie restriction should be considered with caution due to the lack of evidence in humans.

The key during this period of confinement would be a balanced diet comprising all the necessary nutrients, including healthy fats with balanced levels of sugar and cholesterol. During confinement, low-calorie diets should not be recommended, as they are not effective in the long term, and do not provide sufficient energy for a person in this situation of staying at home. Carbohydrates are an appropriate source of energy and are needed daily, mainly if associated with aerobic exercise. Foods rich in carbohydrates with a low glycemic index (whole grains, brown rice, vegetables, legumes, fruits, etc.) and proteins are an essential part of the diet, especially during this period of greater inactivity and we should avoid carbohydrates with a high glycemic index such as sugars, sweets, or bread. Foods rich in proteins with a lower percentage of fat such as chicken and turkey meat, fish, cooked eggs, fresh cheeses, legumes (soy), as well as dairy products such as yogurt and cottage cheese, are recommended because proteins have a stimulating effect on metabolism and are involved in the elimination of fats. Therefore, the combination of an adequately balanced diet and regular physical exercise, should serve to maintain a stable metabolic balance.

### 3.6. Physical Activity to Mitigate the Metabolic Impacts of Confinement

In many countries, an undesirable effect of the COVID-19 pandemic is restricted outdoor physical exercise. Recommendations include indoor walking every 2 h in order to stimulate both cardiovascular and musculoskeletal systems. Aerobic exercise, such as jogging at home if there is enough space, is highly recommended, as well as performing flexo-joint extension (shoulders, elbows, wrists, back, hip, knees, and ankles) and strength, flexibility and stretching exercises of the main muscle groups. A central-question is: what type of training is appropriate for an individual with metabolic syndrome? In a meta-analysis examining the effects of aerobic exercise training, strength training, or both combined, on cardiovascular risk factors in patients with metabolic syndrome, it was observed that aerobic training improved waist circumference, fasting blood glucose, HDL-cholesterol, triglycerides, diastolic blood pressure, and VO_2peak_ [43]. No changes were related to strength training alone, probably due to the limited data available. Accordingly, high-intensity aerobic training performed over more than 12 weeks (3 days/week), shows the most marked effects on cardiovascular risk factors [43]. Inactivity slows the metabolic benefits of exercise, while exercise improves postprandial lipemia levels, glucose tolerance, and insulin sensitivity, all of which are risk factors for CVD. Another study highlights that physical inactivity (e.g., sitting 13.5 h/day and walking fewer than 4000 steps a day) provokes resistance to metabolic improvements that usually result from an acute episode of aerobic exercise, emphasizing that exercise, a heart-healthy diet, and an active lifestyle should be combined to achieve a healthy cardiometabolic profile [44].

Another added problem during this period of home confinement at is that the only form of exercise for many people is walking up and down the corridor of their house. Thus, the question arising is how many steps per day are recommended? Taking more steps per day (8000 vs. 4000 steps per day) is associated with lower all-cause mortality but a significant association has not been found between step intensity and mortality after adjusting for the total number of steps per day [45]. It is essential to walk as much as possible regardless of intensity, since muscle is an endocrine organ that modulates the production of substances according to their activity, requiring minimal muscle activation to obtain benefits [13]. During confinement, it is very likely that physical activity drops drastically possibly resulting in more hours of bed rest, with the consequent loss of muscle mass and its impaired function, and increases in glucose intolerance. Nutrition will play an even more significant role at this challenging moment and must include a consumption in the range of 1.4–2.0 g/kg per day protein to protect from the consequences of muscle inactivity [46,47]. Protein intake should be customized according to different factors, such as the type of population (young or older) [48], energy status, the quality of protein intake or the mode and intensity of exercise [46].

An exercise program for the confinement period has been proposed [49]. Recommendations include increasing the frequency of exercise to 5–7 days per week, 200–400 min of aerobic training and 2–3 days of resistance training. Mobility should be included every day as well as balance and coordination distributed through different training. This should be done at least two times per week. For older people, moderate intensity exercise is recommended during quarantine. Exercise may be performed without any specific training materials. Resistance training can be done through body weight exercises, such as squats, push-ups or sit-ups. Household items such as water bottles or packets of food can be used as weights. Different examples of aerobic exercise are dancing, stair climbs and walking or running on the spot. Balance exercises could be performed via stepping over obstacles or walking along a straight line marked out on the floor [49,50]. Additionally, yoga or traditional Tai Ji Quan can be considered as they do not require any equipment or large space [50].

Resistance training has been demonstrated to reduce the loss of muscle mass and muscle strength [51,52,53] and improve bone density [54], metabolic health and insulin resistance [53]. Resistance training should be properly designed for older adults following principles of individualization, periodization, and progression [53]. However, minimally supervised home-based training has also been shown to be a safe and effective method of increasing body muscle strength [51]. Resistance training can be useful to fight against the metabolic and physical consequences of COVID-19. However, exercise programs should consider other important components: aerobic, balance, coordination and mobility [49].

## 4. Limitations

As the main limitation of the studies reviewed, with the exception of three studies [24,26,28] (n = 45, n = 26, n = 22, respectively), most had <20 participants, so statistical power was low. Participants were mainly males (n = 119) and there was a lower number of females (n = 51). Additionally, none of the studies reported on sex differences, an aspect that would be interesting to explore. Nevertheless, as results exist for both sexes, we believe that recommendations can be followed by both.

Another aspect we should highlight is the fact that not all the articles measured the same variables. Although all examined glycemic control, not all analyzed CRF, body composition, lipid profile or inflammatory factors. These variables could have provided much evidence of the deleterious impact of step reduction. We found no study including a short term step reduction intervention in obese persons or people with metabolic syndrome. According to the high prevalence of both syndromes and their pathogens, it is probably that in these populations, the health impacts of short term physical inactivity will be worse.

In most of the studies reviewed, while physical activity was reduced, participants maintained their dietary habits and, as a result, their energy balance was positive [19,20,24,25,27,28]. Another three articles added overfeeding to the intervention [22,23,26], and just one examined calorie restriction [23]. In these studies, most deleterious consequences were avoided. So it is unknown if the negative repercussions of acute physical inactivity are derived from the inactivity itself, the positive energy balance or from both aspects. According to the current evidence, home exercise as well as a healthy balanced diet avoiding overeating could be a good strategy to mitigate the impacts of acute physical inactivity. Further research on this topic is needed.

## 5. Conclusions

**Figure 2:**
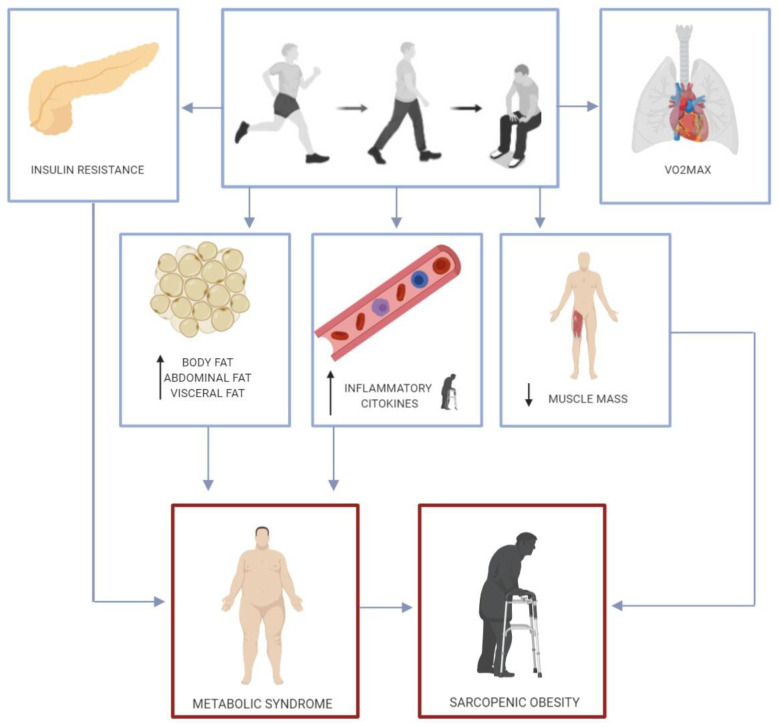
Consequences of a short-term reduction in physical activity.

Metabolic syndrome, also known as “insulin resistance syndrome”, is defined as “a constellation of interconnected physiological, biochemical, clinical, and metabolic factors that directly increases the risk of atherosclerotic CVD, type 2 diabetes mellitus, and all-cause mortality” [55]. Metabolic syndrome is strongly linked to insulin resistance, oxidative stress, inflammation, obesity, endothelial dysfunction and CVD [56]. In turn, physical inactivity has been related to every described risk factor for metabolic syndrome: dyslipidemia, hypertension, hyperglycemia, visceral obesity, and prothrombotic and proinflammatory events (Figure 2).

Insulin resistance is a central component of metabolic syndrome [56] and while high levels of daily physical activity can prevent insulin resistance, physical inactivity is a primary cause of insulin resistance and a loss of insulin sensitivity in skeletal muscle [2]. In the studies reviewed, it was observed that just two weeks of physical inactivity and a positive energy balance can increase insulin resistance and modify glycemic control [19,20,21,22,23,24,25,26,27,28]. Obesity and visceral obesity are also central components of metabolic syndrome [55,56] with an important role in CVD [57] through different mechanisms such as insulin resistance and the induction of a proinflammatory state [58]. The studies reviewed detected increases in body fat [22,23,24,27] and abdominal fat mass [19,22,24] after just one or two weeks of step reduction associated with a positive energy balance.

The role of inflammation in the pathogenesis of metabolic syndrome and CVD has been well documented [55,56,59]. In the studies conducted in elderly subjects reviewed here, it was confirmed that two weeks of physical inactivity led to increases in TNF-α, IL6 and CRP [27,28]. TNF-α and IL-6 are cytokines with endocrine, autocrine and paracrine functions, and their gene expression is increased in the adipocytes, macrophages and lymphocytes of obese individuals [56]. TNF-α acts locally on adipocytes and reduces insulin sensitivity via different mechanisms, increases FFA levels through the induction of lipolysis, and inhibits adiponectin release [60]. This cytokine also attenuates nitric oxide-mediated vasodilation and is involved in the vascular pathology of metabolic syndrome, atherosclerosis and coronary disease [56]. IL-6 creates insulin resistance in the liver and enhances the hepatic synthesis of acute phase proteins such as CRP and fibrinogen. CRP shows high correlation with metabolic syndrome, diabetes and CDV, and fibrinogen leads to a prothrombotic state [56,60]. IL-6 also promotes the expression of adhesion molecules by endothelial cells and activates the local renin-angiotensin system, whose activation contributes to metabolic syndrome development [56,60].

Acute sedentarism can be deleterious for health though other mechanisms. It has been reported that short term physical inactivity lowers VO_2max_ [20,22,23,24] and also reduces lean mass and fat free mass, with greater impacts on the lower body [19,20,24,27]. Both low skeletal muscle mass and maximal aerobic capacity (VO_2max_) are biomarkers associated with a shorter life expectancy [2]. The impact of this reduction in muscle mass could be especially important in the elderly, due to a higher prevalence of sarcopenia and its health impacts in these subjects. Moreover, sarcopenia combined with obesity (sarcopenic obesity) has been linked to a worse metabolic impact and increased risk of mortality [61].

More research is needed to examine whether energy restriction could avoid the consequences of acute physical inactivity as suggested in one of the articles reviewed [23]. Nevertheless, there is evidence to support that at lower levels of physical activity, energy intake is dysregulated leading to a positive energy balance [35,36,37,38]. Maintaining food glycemic control is a specific measure in patients with diabetes to help reduce infection risk and severity. In effect, it has been also recommended that attention be paid to nutrition and adequate protein intake, along with exercise to improve immunity [62]. Short-term physical inactivity and a positive energy balance can have several consequences for health related to reduced insulin sensitivity, higher total body and central fat, and a proinflammatory state, which are all central risk factors for metabolic syndrome. For the elderly, consequences could be worse, increasing the risk of developing sarcopenic obesity.

According to recent evidence, adequate control of metabolic disorders is important to reduce the risk of severe COVID-19. We should try to avoid the deleterious consequences of physical inactivity and positive energy balance by maintaining physical activity and exercise levels in a safe home environment and adhering to a healthy diet. Of course, this is also important for people without metabolic disorders to avoid the reported deleterious effect of physical inactivity and positive energy balance, which may prompt the development of metabolic syndrome and its comorbidities. COVID-19 varies from a mild self-limiting flu-like illness to full-blown pneumonia, respiratory failure and death [62]. Hypertension, diabetes, and CVD have been identified as potential risk factors for the more severely ill patients. In addition, COVID-19 could also enhance damage to the heart in patients with CVD [63,64]. As obesity increases, so does risk of chronic disease related to metabolic syndrome and based on recent data, obese individuals are also being considered at high risk for severe complications of COVID-19 [65,66].

Accordingly, our recommendations during this period of confinement are to avoid overeating by following a healthy balanced diet. This diet should be based on carbohydrates with a low glycemic index, such as vegetables, legumes or fruits, healthy fats and food rich in proteins with a lower percentage of fat. Moreover, calorie intake may be restricted due to reduced activity or physical inactivity. This nutrition recommendation should be combined with an adequate daily physical activity program designed by sports science specialists to prevent metabolic-related health problems. This program should consider different components: resistance, aerobic, mobility, coordination and balance. The recommended frequency of training is 5–7 days per week, including at least 2–3 days of resistance training. 

Once COVID-19 transmission is controlled, different countries are setting new regulations in which exercising in the street is allowed. Each set of regulation may have different rules, in terms of time, or number of people training together. Depending on the country, personal training or in small groups might be permitted. However, reopening of gym or sport centers to the population at large could take longer. For this reason, resistance training should continue at home as recommended here, as well as exercise for mobility, coordination and balance. As indicated by the studies reviewed in this article, short term physical inactivity can lead to a reduction in CFR and also in muscle mass. In some countries this inactivity has been much longer. Accordingly, people must consider that their fitness level is lower than before confinement if they begin to exercise in the street, such as running. Counselling by a sports science specialists could be useful to avoid injuries. Likewise, it is imperative that all the actions we carry out comply with the social distancing recommended by the health authorities.

## Figures and Tables

**Table 1:** Summary of the studies reviewed examining the effects of acute physical inactivity and/or overfeeding.

Study	Subjects	Intervention	Blood Parameters	Inflammatory Parameters	Lipid Profile	Body Composition	CRF
**Olsen et al. 2014** [19]	8 healthy men <2 h EX/week, >3500 steps/day 27.1 (5.7) years BMI 22.9 (4.0) kg/m^2^	3-weeks SR 6203 (5135–7271) to 1394 (1261–1528) steps/day Maintained dietary habits	OGTT ↑ insulin AUC	Not reported	Not reported	Not reported	Not reported
	10 healthy men <2 h EX/week, >3500 steps/day 23.8 (4.6) years BMI 22.1 (2.1) kg/m^2^	2-week SR 10,501 (8755–12,247) to 1344 (1272–1416) steps/day Maintained dietary habits	OGTT ↑ insulin AUC, C-peptide levels OFTT ↑ insulin, C-peptide, TG	Not reported	Not reported	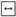 FM ↑ intra-abdominal fat ↓ fat free mass	Not reported
**Krogh-Madsen et al. 2010** [20]	10 healthy men <2 h EX/week, >3500 steps/day 23.8 (1.5) years BMI 22.1 (0.7) kg/m^2^	2-week SR 10,501 (808) to 1344 (33) Maintained dietary habits	↓ GIR during the clamp ↓ peripheral insulin sensitivity (H-E) ↓ insulin provoked muscle Akt phosphorylation 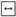 hepatic glucose production, plasma glucose, insulin, C-peptide	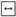 TNF, IL-6, IL-15, adiponectin, leptin	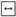 TG, FFA	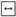 FM ↓ BM, leg lean mass 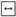 trunk and arm lean mass	↓ VO_2max_
**Mikus et al. 2012** [21]	12 healthy participants (4 F, 8 M) >10000 steps/day 29 (1) years BMI 23.6 (0.9) kg/m^2^	3-day SR 12,956 (769) to 4319 (256) steps/day Maintained dietary habits	CGM ↑ post-prandial glucose, fasting insulin 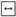 pre-meal blood glucose, 24 h average glucose OGTT ↑ insulin AUC, C-peptide, HOMA-IR 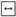 glucose AUC ↓ Matsuda	Not reported	Not reported	Not reported	Not reported
**Dixon et al. 2013** [25]	18 healthy men EX > 30 min/ 5 d/week 9 overweight: 49 (1.0) years BMI 29.3 (1.2) kg/m^2^ 9 lean men: 51.5 (1.4) years BMI 29.3 (1.2) kg/m^2^	1-week SR <4000 steps/day Maintained dietary habits	OGTT ↑ insulin AUC, glucose AUC Overweight: >insulin AUC, glucose AUC	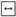 CRP, IL-6, TNF-α, WBC, sICAM, ALT Overweigh: >CRP, ALT	↑ TG 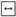 FFA, TC, HDL-c, LDL-c Overweight: >FFA, TG	Not reported	Not reported
**Bowden-Davies et al. 2018** [24]	45 healthy participants (28 F, 17 M) >10,000 steps/day, >2 h EX/week 16 FDR+ve: 40(14) yearsBMI 27 (5) kg/m^2^ 29 FDR-ve: 33 (13) years BMI 24 (3) kg/m^2^	2-week SR <1500 steps/day (mean decreased: 10,285) Maintained dietary habits	OGTT ↑ insulin AUC, glucose AUC ↓ Matsuda, muscle insulin sensitivity 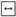 hepatic insulin resistance, NEFA AUC FDR+ve: <insulin sensitivity (after SR and resuming of PA)	Not reported	↑ TC, LDL-c, TG	↑ FM. liver fat ↓ total lean mass, lower limb lean mass 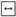 arm lean mass FDR+ve: >increase of android fat	↓ VO_2max_
		2-week resumed usual PA	
**Knudsen et al. 2012** [22]	9 healthy men >10,000 steps/day 24.3 (3.3) years BMI 21.6 (2.5) kg/m^2^	2-week SR + overfeeding 10,278 (2399) to 1521 (488) steps/day 2762 (299) kcal to 4197 (290) kcal	OGTT ↑ insulin AUC (day 7, day 14), Matsuda (day 3, day 7) 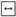 glucose AUC ↓ Peripheral insulin sensitivity (H-E) 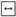 plasma glucose, C-peptide, hepatic glucose production	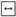 TNF-α, IL-6 ↑ leptin, adiponectin	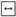 FFA, TG	↑ BM, FM, android and visceral fat 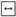 FFM	↓ VO_2max_
		16-day resumed usual PA	Returned to baseline			Remained elevated	Returned to baseline
**Winn et al. 2019** [23]	10 healthy participants (4 F, 6 M) >90 min PA 3 days/week and >10,000 steps/day 24 (1) years BMI < 28 kg/m^2^	10-day SR (>10,000 to <50,000 step/day) + control diet (400 kcal/day deficit) 4-week washout 10-day SR + higher protein diet (400 kcal/day deficit)	OGTT 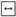 Postprandial glucose, insulin, NEFA, 2-h glucose and 2-h insulin, C-peptide, hepatic insulin extraction, plasma glucose and insulin	Not reported	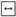 TG, LDL-c, oxidized LDL ↓ HDL-C, TC	↓ BM abdominal FM 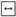 FFM, FM	↓ VO_2max_
		10-day SR + overfeeding (880 kcal/d)	OGTT ↑ HOMA-IR, 2h-glucose, 2h-insulin 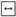 glucose AUC, NEFA ↑ plasma glucose, insulin, C-peptide 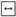 hepatic insulin extraction	Not reported	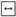 TG, TC, HDL-c, LDL-c, oxidized LDL	↑ BM, FM 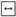 FFM	↓ VO_2max_
**Walhin et al. 2013** [26]	26 healthy men Vigorous-intensity EX > 30 min/ 3 day/week 25 (7) years BMI 23.8 (2.5)	1-week SR: 12,562 (3520) to 3762 (860) + overfeeding (+50% kcal) + 45 min/day treadmill running at 70% VO_2max_	OGTT 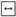 Matsuda, B- cell function, insulin AUC	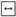 ALT, CRP, IL-6, WBC	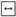 HDL-C. LDL-C, NEFAs, TG, CT	↑ BM, waist/hip circumference, lean mass 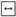 FM	Not reported
		1-week SR: 10,544 (2756) to 3690 (400) + overfeeding (+50% kcal) + not training	↓ Matsuda, B- cell function ↑ insulin AUC OGTT	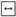 ALT, CRP, IL-6 ↑ adiponectin, WBC	↑ CT 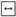 HDL-C. LDL-C, NEFAs, TG	↑ BM, waist/hip circumference, lean mass 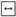 FM	Not reported
**Breen et al. 2013** [27]	10 healthy older adults (5 F, 5 M) >3500 steps/day 72.3 (1.0) years; BMI 29.0 (1.8) kg/m^2^	2-week SR: 5962 (695) to 1413 (110) steps/day Maintained dietary habits	OGTT ↑ HOMA-IR, glucose AUC and AUC ↓ Matsuda 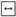 C-peptide AUC	↑ TNF-α, CRP 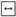 IL-6	Not reported	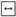 total FM, FFM ↓ leg FFM ↑ % FM ↓ MPS 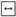 isometric MVC, SPPB	Not reported
**McGlory et al. 2018** [28]	22 healthy older adults >3500 steps/day 12 M: 69 (3) years; BMI 27.3 (4.6) 10 F: 70 (5]) years; BMI 27.7 (5.1)	2-week SR: 7362 (3294) to 991 (97) steps/day Maintained dietary habits 2-week resumed usual PA	OGTT ↑ glucose and insulin AUC, HOMA-IR ↓ Matsuda	↑ TNF-α, CRP, IL-6	Not reported	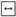 BMI, %total FM, lean mass ↓ MPS 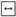 isometric MVC	Not reported

Area under curve (AUC), alanine transaminase (ALT), body mass (BM), body mass index (BMI), cardiorespiratory fitness (CRF), continuous glucose monitoring (CGM), C-reactive protein (CRP), exercise (EX), fat mass (FM), females (F), first-degree relatives with type 2 diabetes (FDR+ve), first-degree relatives without type 2 diabetes (FDR−ve), free fatty acids (FFA), glucose infusion rate (GIR), high density lipoprotein cholesterol (HDL-c), homeostatic model assessment for insulin resistance (HOMA-IR), hyperinsulinemic-euglycemic during clamp (H-E), LDL-C (low-density lipoprotein cholesterol), males (M), maximal voluntary contraction (MVC), muscle protein synthesis (MPS), non-esterified fatty acids (NEFA), oral glucose tolerance test (OGTT), physical activity (PA), short physical performance battery (SPPB), soluble intercellular adhesion molecule (sICAM), step reduction (SR), triglycerides (TG), total cholesterol (TC), tumor necrosis factor alpha (TNF-α), white blood cells (WBC), 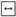 no significant change, ↑ significant increase, ↓ significant decrease, > significantly higher, < significantly lower.
